# Oncological Outcomes for Patients Harboring Positive Surgical Margins Following Radical Cystectomy for Muscle-Invasive Bladder Cancer: A Retrospective Multicentric Study on Behalf of the YAU Urothelial Group

**DOI:** 10.3390/cancers14235740

**Published:** 2022-11-22

**Authors:** Gautier Marcq, Luca Afferi, Yann Neuzillet, Timo Nykopp, Charlotte S. Voskuilen, Marc A. Furrer, Wassim Kassouf, Atiqullah Aziz, Anne Sophie Bajeot, Mario Alvarez-Maestro, Peter Black, Morgan Roupret, Aidan P. Noon, Roland Seiler, Kees Hendricksen, Mathieu Roumiguie, Karl H. Pang, Paul Laine-Caroff, Evanguelos Xylinas, Guillaume Ploussard, Marco Moschini, Paul Sargos

**Affiliations:** 1 Division of Urology, McGill University Health Centre, McGill University, 1001 Decarie Blvd, D02.7210, Montreal, QC H4A 3J1, Canada; 2 Urology Department, Claude Huriez Hospital, CHU Lille, F-59000 Lille, France; 3 CNRS, Inserm, CHU Lille, Institut Pasteur de Lille, UMR9020-U1277—CANTHER—Cancer Heterogeneity Plasticity and Resistance to Therapies, University Lille, F-59000 Lille, France; 4 Department of Urology, Luzerner Kantonsspital, 6000 Luzern, Switzerland; 5 Department of Urology, Foch Hospital, Paris-Saclay—UVSQ University, 92150 Suresnes, France; 6 Department of Surgery, Kuopio University Hospital and University of Eastern Finland, PL 100, 70029 Kuopio, Finland; 7 The Netherlands Cancer Institute—Antoni van Leeuwenhoek Hospital, Department of Urology, 1066 CX Amsterdam, The Netherlands; 8 Department of Urology, University Hospital of Bern, University of Bern, 3012 Bern, Switzerland; 9 Department of Urology, The Royal Melbourne Hospital, Parkville 3050, Australia; 10 Department of Urology, The Guy's Hospital, London SE1 9RT, UK; 11 Department of Urology, München Klinik Bogenhausen, 81925 Munich, Germany; 12 Department of Urology, ICUT CHU, 31300 Toulouse, France; 13 Department of Urology, Hospital Universitario La Paz, 28046 Madrid, Spain; 14 Vancouver Prostate Centre, Department of Urologic Sciences, University of British Columbia, Vancouver, BC V6H 3Z6, Canada; 15 Sorbonne University, GRC 5 Predictive Onco-Uro, AP-HP, Urology, Pitie-Salpetriere Hospital, F-75013 Paris, France; 16 The Department of Urology, Sheffield Teaching Hospitals NHS Foundation Trust, Sheffield S10 2JF, UK; 17 Department of BioMedical Research, University of Bern, 3012 Bern, Switzerland; 18 Department of Oncology and Metabolism, Academic Urology Unit, University of Sheffield, Sheffield S10 2TN, UK; 19 Department of Urology, Bichat-Claude Bernard Hospital, Assistance Publique-Hopitaux de Paris, Paris University, 75018 Paris, France; 20 Department of Urology, La Croix du Sud Hospital, 31130 Quint Fonsegrives, France; 21 Department of Urology and Division of Experimental Oncology, Urological Research Institute, IRCCS San Raffaele Scientific Institute, 20132 Milan, Italy; 22 Department of Radiotherapy, Institut Bergonie, 33076 Bordeaux, France

**Keywords:** bladder neoplasm, margins, relapse, survival, chemotherapy, radiotherapy

## Abstract

**Simple Summary:**

In this study, we investigated the oncological outcomes of muscle-invasive bladder cancer patients harboring positive surgical margins following radical surgery. We found that half of them will recur within a year after treatment and will eventually die of the disease. We observed a potential role for adjuvant chemotherapy in this patient population, but clinical trials are needed in this population.

**Abstract:**

Introduction: Adjuvant therapy has no defined role for patients with positive surgical margins (PSMs) following radical cystectomy (RC) for muscle-invasive bladder cancer (MIBC). The aim of our study was to describe loco-regional recurrence-free survival (LRFS), metastatic-free survival (MFS), recurrence-free survival (RFS), cancer-specific survival (CSS) and overall survival (OS) and identify predictors of each endpoint in patients with PSMs following RC for MIBC. Methods: A collaborative retrospective cohort study was conducted on 394 patients with PSMs who underwent RC for MIBC between January 2000 and December 2018 at 10 tertiary referral centers. Patients receiving perioperative radiotherapy were excluded from the study. Kaplan–Meier curves were used to estimate patient survival. Cox regression analysis was used to identify predictors of survival. Results: Median age at surgery was 70 years (IQR 62–76) with 129 (33%) and 204 (52%) patients had pT3 and pT4 tumors, respectively. Nodal metastasis (pN+) was identified in 148 (38%). Soft tissue PSMs were found in 283 (72%) patients, urethral PSMs in 65 (16.5%), and ureteral PSMs were found in 73 (18.5%). The median follow-up time was 44 months (95% CI 32–60). Median LRFS, MRFS, RFS, CSS, and OS were 14 (95% CI 11–17), 12 (95% CI 10–16), 10 (95% CI 8–12), 23 (95% CI 18–33), and 16 months (95% CI 12–19), respectively. On multivariable Cox regression analysis, the pT3–4 stage, pN+ stage, and multifocal PSMs were independent predictors of LRFS, MRFS, RFS, and OS. Adjuvant chemotherapy improved all oncological outcomes studied (*p* < 0.05). The number of lymph nodes removed was independently associated with better LRFS, MRFS, and RFS. Advanced age at diagnosis was independently associated with worse OS. Conclusion: Patients with PSMs following RC have poor outcomes since half of them will recur within a year and will die of their disease. Among all PSMs types, patients with multifocal PSMs harbor the worst prognosis. We observed a benefit of adjuvant chemotherapy, but clinical trials evaluating innovative adjuvant strategies for these patients remain an unmet need.

## 1. Introduction

The incidence of positive surgical margins (PSMs) following radical cystectomy (RC) for muscle invasive bladder cancer (MIBC) varies widely in the literature, ranging from 4% to 15% [1,2,3,4,5,6,7]. The prognostic value of PSMs could be related to their location and their multifocality (1, 2). The presence of PSMs impacts oncological outcomes negatively [8]. However patients with PSM have been excluded from most trials investigating the role of adjuvant chemotherapy and immunotherapy [9]. In the most recent trials testing adjuvant immunotherapy, IMvigor010 [10] and CheckMate 274 [11] excluded these patients. However, AMBASSADOR (NCT03244384), the results of which have not yet been reported, allowed them to be enrolled. Since these patients were also excluded from trials testing adjuvant chemotherapy, and since adjuvant radiation therapy (RT) is still investigated in randomized trials [8], there is no defined role for adjuvant therapy in these patients.

Most of available studies have reported only a limited number of patients harboring PSMs following RC and have compared their outcomes to patients with negative surgical margins. We aimed to focus on a larger population of only patients with PSMs and to describe their oncological outcomes, including loco-regional recurrence-free survival (LRFS), metastatic-free survival (MFS), recurrence-free survival (RFS), cancer specific survival (CSS), and overall survival (OS) in a large multi-institutional dataset.

## 2. Methods

### 2.1. Study Design

This was a retrospective cohort study performed at 10 academic centers worldwide. Data from patients with MIBC receiving RC between January 2000 and December 2018 and found to have PSMs were reviewed. Institutional review board approval was obtained for each center and data-sharing agreements were exchanged.

### 2.2. Patient Selection and Collected Information

We included patients with a pathological confirmation of urothelial MIBC with PSMs who underwent RC at each center. We excluded patients with metastatic disease at the time of RC (defined by non-regional lymph nodes or distant metastasis) and patients with no follow-up information. In addition, patients who underwent adjuvant RT were also excluded as we aimed for an unbiased cohort in which predictive factors for relapse could be identified as per the standard of care management. Basic demographic characteristics were obtained, such as age at diagnosis, gender, history of non-MIBC, and clinical tumor and nodal stages. Types of surgical approach (including extent of lymph node dissection), pathological tumor and nodal stages, number of lymph nodes removed, number of positive lymph nodes, and the presence of concomitant carcinoma in situ (CIS) in the RC specimen were also collected. Pathological margins (defined by the presence of invasive or non-invasive cancer at the inked surface) were detailed as uni- or multifocal, and soft tissue, urethral, or ureteral margins. All surgical specimens were processed according to standard pathologic procedures at each institution by a uropathologist.

The type of adjuvant therapy was stratified between observation or chemotherapy. Patients were followed lifelong according to each center’s convention with physical examination, serum chemistry evaluation, serial cross-sectional imaging, and cystoscopy when relevant.

### 2.3. Endpoints

For all subsequent time-to-event outcomes, the follow-up period started with the day of RC, and patients were uncensored at event and censored until the last follow-up day. Loco-regional recurrence was defined as any pelvic cancer recurrence (cystectomy bed, pelvic lymph-nodes up to aortic bifurcation, or soft tissue sites) or death from bladder cancer. Metastatic recurrence was defined as any distant metastasis or death from bladder cancer. Recurrence was, therefore, defined as any recurrence or death from bladder cancer (pooling loco-regional and metastatic recurrences). De novo urothelial carcinoma in the upper tract was not considered as recurrence unless the recurrence was seen at the ureteral PSMs site. Finally, the cause of death was identified, distinguishing cancer-specific (CSS) or overall survival (OS).

### 2.4. Statistical Analysis

Categorical variables were reported as frequencies and proportions; continuous variables were reported as median and interquartile range (IQR). The Kaplan–Meier method was used to estimate LRFS, MRFS, RFS, CSS, and OS. Kaplan–Meier curves were stratified by positive margin status (multifocal, urethral, ureteral, and soft tissue) and tested for differences with the log-rank test. The effects of patient demographics and clinical and pathological variables on outcomes were estimated through a stepwise Cox multivariable regression model for the variable with a *p* value < 0.2 in univariate analysis and with less than 20% missing values. Log linearity hypotheses were verified for quantitative variables and proportional hazard assumptions were verified for all variables. All statistical analyses were performed using SAS 9.4 (SAS Institute; Cary, NC, USA). All *p*-values < 0.05 were considered statistically significant.

## 3. Results

### 3.1. Patient Characteristics

A total of 394 patients with PSMs were identified, of whom 319 (81%) were males and 75 (19%) were females. Patient demographics and clinical and pathological features are summarized in Table 1. Median age at surgery was 70 years (62–76). Open RC was performed in 322 (82%) patients, and 195 (50%) patients had an ileal conduit as a urinary diversion. On RC final pathology, 129 (33%), 204 (52%), and 148 (38%) patients were pT3, pT4, and pN+, respectively.

Soft tissue PSMs were identified in 283 (72%), urethral PSMs in 65 (16.5%), ureteral PSMs in 73 (18.5%) and multifocal PSMs in 63 (16%) cases. In patients with multifocal PSMs, 47 (75%) had at least 1 soft tissue PSM (25 had multifocal soft tissue PSMs, 12 had ureteral and soft tissue PSMs, 7 had urethral and soft tissue PSMs, and 3 had ureteral, urethral, and soft tissue PSMs.

Only 31 patients (8%) received neoadjuvant chemotherapy. Adjuvant chemotherapy was administered in 69 (17.5%) patients.

### 3.2. Survival Analyses

Loco-regional and metastatic recurrence was observed in 215 (55%) and 219 (56%) patients, respectively. When excluding death from bladder cancer as an event, loco-regional and metastatic recurrence was observed in 110 (28%) and 155 (39%) patients, respectively. During follow-up, 243 (62%) had recurred (pooling loco-regional and metastatic recurrences) and 181 (46%) had died from bladder cancer. We recorded 238 (60%) deaths from any cause. Oncological outcomes are summarized in Table 2. The median follow-up time for the entire cohort was 44 months (95% CI 32–60).

**Figure 1:**
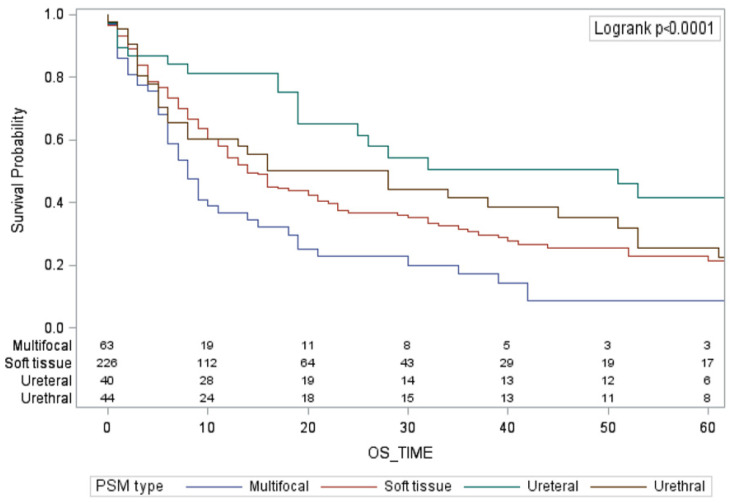
Kaplan–Meier curves reporting overall survival probability stratified by the type of PSMs.

Median LRFS, MFS, RFS, CSS, and OS were 14 (95% CI 11–17), 12 (95% CI 10–16), 10 (95% CI 8–12), 23 (95% CI 18–33), and 16 months (95% CI 12–19), respectively (Table 2). Multifocal PSMs and soft tissue PSMs were associated with an increased risk of death from any cause compared to other PSMs types. Median OS was 8 months (95% CI 12–19), 12 months (95% CI 10–16) and 15 months (95% CI 6–30) for patients with multifocal PSMs, soft tissue PSMs, and urethral PSMs, respectively, compared to 28 months (95% CI 18–51) for patients with ureteral PSMs (*p* < 0.001, Figure 1).

### 3.3. Predictive Factors for Survival in Uni- and Multivariable Analyses

On univariate Cox regression analysis, compared to soft tissue PSMs, ureteral PSMs were associated with better OS (HR = 0.51, 95% CI 0.31–0.84, *p* = 0.0078), whereas urethral PSMs (HR = 0.87, 95% CI 0.59–1.30, *p* = 0.500) were not. The presence of multifocal PSMs (HR = 1.66, 95% CI 1.19–2.31, *p* = 0.0027) was associated with worse OS compared to soft tissue PSMs.

On multivariable Cox regression analysis, pT3–4 stage, pN+ stage, and multifocal PSMs were independent predictors for worse MFS, RFS, and OS (Table 3). The use of adjuvant chemotherapy improved all oncological outcomes studied (*p* < 0.05). The number of lymph nodes removed was independently associated with improved LRFS, MFS, RFS, but not OS, whereas higher age at diagnosis was independently associated with worse OS, but not LRFS, MFS, or RFS. 

## 4. Discussion

In this large multi-institutional retrospective study, we have shown that among patients harboring PSMs following RC for MIBC, patients with multifocal PSMs, a lower number of LN removed at the time of RC, and advanced pathological tumor and nodal stages had significantly worse oncological outcomes. Moreover, the use of adjuvant chemotherapy significantly improved these outcomes. We were not able to identify different predictive factors for loco-regional or metastatic recurrence that may help to tailor adjuvant therapy based on the type of PSMs.

To our knowledge, this study represents the largest report investigating patients harboring PSMs following RC. The previous largest report was also an international multi-institutional retrospective report including 278 patients (median follow-up 37 months) [12]. In their study, the authors reported that soft tissue PSM was an independent predictor of disease recurrence and cancer-specific mortality (HR 1.52 and HR 1.51, *p* < 0.001, respectively) with a median time to cancer specific mortality of 20 months. Our analysis corroborates those findings, showing a median OS of 12 months (95% CI 10–16) in this population. Interestingly they also reported that adjuvant chemotherapy was significantly associated with better cancer-specific mortality (without providing the HR) and stressed that PSMs should be reported in every RC pathological report. Xylinas et al. also reported a large multi-institutional retrospective study on 231 patients with soft tissue PSMs with a median follow-up (FU) of 37 months [1]. They also confirmed poor outcomes of patients with soft tissue PSMs, however, 20% of their patients with soft tissue PSMs did not experience disease recurrence and 25% were alive at 5 years. In our analysis the 5-year RFS was 16% (95% CI 11–23) and the 5-year OS was 18% (95% CI 12–25), depicting higher risk of recurrence and death compared to Xylinas et al. Neuzillet et al. have also reported a retrospective multicenter analysis with a median FU of 24 months, including 154 patients with all types of PSMs [2]. Similar to our analysis, they did not identify specific factors able to predict the type of recurrence (local vs metastatic).

The number of lymph nodes removed at the time of RC has impacted LRFS, MFS, and RFS in this study, highlighting the dramatic importance of the initial lymph node dissection at the time of RC (although we failed to prove a negative impact on OS in our cohort). Even if the extent of the lymph node dissection is still under debate [13,14], a subset analysis of the SWOG 8710 by Herr et al. showed that an adequate lymph node dissection with ≥10 nodes removed was associated with an OS benefit regardless of receipt of neoadjuvant chemotherapy [15], in line with our results (a median of 11 lymph nodes removed in our analysis). Moreover, the number of lymph nodes removed is associated with loco-regional recurrence rates; a risk stratification approach to identify patients at highest risk for recurrence has been developed, including pathological tumor and nodal stages as well as PSMs, corroborating our results [16].

In this study, we have identified an OS benefit with the use of adjuvant chemotherapy (HR = 0.6 95% CI 0.4–0.8, *p* = 0.003) for patients harboring PSMs following RC. Randomized controlled trials (RCTs) investigating the role of adjuvant chemotherapy have excluded patients with PSMs [17,18,19,20,21]. However, in a meta-analysis pooling the results of adjuvant chemotherapy trials (including 945 patients from 9 RCTs) [20], an OS benefit was observed, and the pooled hazard ratio across all 9 trials was 0.77 (95% CI, 0.59–0.99; *p* = 0.049). It is important to note that this meta-analysis is limited since none of the included trials had fully accrued and individual patient data were not used in the final analysis. More recently, the Advanced Bladder Cancer Meta-analysis Collaborators Group investigated again the role of adjuvant chemotherapy in a systematic review and meta-analysis of individual participant data from 10 randomized trials [22]. Out of the 10 included trials, 3 mentioned excluding patients with PSMs, while the information was not reported in the other trials. Adjuvant cisplatin-based chemotherapy improved OS (hazard ratio [HR] = 0.82, 95% confidence interval [CI] = 0.70–0.96, *p* = 0.02), RFS (HR = 0.71, 95% CI = 0.60–0.83, *p* < 0.001), LRFS (HR = 0.68, 95% CI = 0.55–0.85, *p* < 0.001), and metastasis-free survival (HR = 0.79, 95% CI = 0.65–0.95, *p* = 0.01). Our finding of OS benefit with the use of adjuvant chemotherapy in a population of patients with PSMs raises the question of its use in clinical practice, especially when taking into account the poor OS reported in our cohort (median OS 16 months), acknowledging, however, the inherent selection bias due to our study design.

### 4.1. Future Perspectives

Many patients are not candidates for adjuvant chemotherapy due to post-operative morbidity [20], pre-existing medical illness, impaired kidney function, or prior exposure to neoadjuvant chemotherapy. Adjuvant immunotherapy with nivolumab has become an option for these patients based on the disease-free survival benefit shown in the Checkmate 274 phase III randomized trial [11], even though IMvigor010 (with atezolizumab) was a negative trial [10]. It is critical to highlight, however, that patients with PSM were excluded from these trials. Moreover, patient selection for adjuvant therapies is key. Biomarkers are eagerly awaited to help clinical practice [23,24].

In light of the high rates of loco-regional recurrence following RC in patients harboring PSMs, as well as locally advanced disease, adjuvant RT could be of interest. There is promising evidence that adjuvant RT can significantly reduce loco-regional recurrences, and that this improvement may translate in better MFS and OS, with acceptable toxicity [25,26]. Clinical trials are ongoing, and accrual to these trials is key. These studies will contribute important data that will help us to better understand the role of RT in locally advanced bladder cancer, including patients with PSMs for whom no adjuvant standard strategy is yet defined.

### 4.2. Limitations

Besides the retrospective design, our study has several limitations that need to be described. Only 8% of the included patients underwent neoadjuvant chemotherapy compared to higher rates that are seen in more contemporary series [27]. This low rate may be explained by the wide inclusion period over which the standard-of-care has evolved with time. Moreover, neoadjuvant chemotherapy may help to reduce the incidence of PSMs so that there is an inherent selection for a lower rate of its use when focusing on patients with PSMs. It is conceivable that PSMs may have an even greater significance in patients who received pre-operative chemotherapy, since the likelihood of salvage with post-operative chemotherapy is reduced. Lymphovascular invasion is a well-known predictive factor of survival following RC [28], but was not available in our dataset. Similarly, we were able to provide neither a soft tissue margin location analysis nor a mapping of loco-regional recurrences, which could be of interest when investigating the role of adjuvant RT in this setting [29]. We also have to acknowledge the lack of a pathology central review as a limitation. Finally, our results showing a potential benefit with the use of adjuvant chemotherapy could be driven by selection bias due to our study design.

## 5. Conclusions

Patients with PSMs following RC have poor outcomes, with half recurring within one year following surgery, most of whom will subsequently die of their disease. Among all PSMs types, patients with multifocal PSMs harbor the worst prognosis. We were unable to identifying predictive factors to distinguish between local and metastatic recurrences. Adjuvant chemotherapy may play a role and should be discussed on a case-by-case basis. Clinical trials evaluating innovative strategies, such as RT and/or immunotherapy for these patients, are an unmet need.

## Figures and Tables

**Table 1:** Patient demographics and clinical and pathological features.

	Total	
	N or Median	% or IQR
	N = 394	
**Gender**		
Male	319	81.0%
Female	75	19.0%
**Median age at diagnosis, years**	70	62–76
**Previous history of NMIBC**		
None	270	68.5%
Yes	116	29.4%
Missing	8	2.0%
**Tumor histology**		
UC	369	93.7%
Squamous	7	1.8%
Adenocarcinoma	2	0.5%
Small cell	3	0.8%
Micropapillary	3	0.8%
Other	10	2.5%
**Clinical tumor stage**		
cT1	0	0%
cT2	290	73.6%
cT3	39	9.9%
cT4	32	8.1%
Missing	33	8.4%
**Clinical nodal stage**		
cN0	265	67.3%
cN1	28	7.1%
cN2	21	5.3%
cN3	3	0.8%
Missing	77	19.5%
**Neoadjuvant chemotherapy**		
No	358	90.9%
Yes	31	7.9%
Missing	5	1.3%
**Surgical approach**		
Open	322	81.7%
Robotic	30	7.6%
Laparoscopic	19	4.8%
Missing	23	5.9%
**Urinary diversion**		
Neobladder	38	9.6%
Ileal conduit	195	49.5%
Continent cutaneous diversion	8	2.0%
Cutaneous ureterostomy	151	38.3%
Missing	2	0.5%
**Type of lymph nodes dissection**		
None	70	17.8%
Standard	248	62.9%
Extended	69	17.5%
Missing	7	1.8%
**Median number of lymph nodes removed**	11	(3–22)
Missing	4.6%	
**Pathologic staging at RC**		
pT0	11	2.8%
pT1	4	1.0%
pT2	42	10.7%
pT3	129	32.7%
pT4	204	51.8%
pTis	4	1.0%
**Concomitant carcinoma in situ at RC**		
No	170	43.1%
Present	103	26.1%
Missing	121	30.7%
**Pathologic lymph node staging at RC**		
pNx	72	18.3%
pN0	167	42.4%
pN1	36	9.1%
pN2	87	22.1%
pN3	25	6.4%
Missing	7	1.8%
**Multifocal PSMs**		
No	331	84.0%
Yes	63	16.0%
**Soft tissue PSMs**		
No	105	26.6%
Yes	283	71.8%
Missing	6	1.5%
**Urethral PSMs**		
No	323	82.0%
Yes	65	16.5%
Missing	6	1.5%
**Ureteral PSMs**		
No	277	70.3%
Yes	73	18.5%
Missing	44	11.2%

Abbreviations are as follows: UC, urothelial carcinoma; RC, radical cystectomy; PSMs, positive surgical margins. Several sums of percentages might not be equal to 100 after rounding the numbers after the decimal point.

**Table 2:** Kaplan–Meier Estimation.

	Local-RFS		MFS		RFS		CSS		OS	
Time from RC (Months)	%	(95% CI)	%	(95% CI)	%	(95% CI)	%	(95% CI)	%	(95% CI)
**Overall Population**										
12	52%	(46–58)	50%	(44–55)	44%	(38–49)	64%	(58–69)	55%	(49–60)
24	38%	(32–44)	38%	(32–43)	31%	(26–37)	49%	(43–55)	40%	(34–45)
36	31%	(26–38)	30%	(24–36)	24%	(19–30)	42%	(35–48)	33%	(27–38)
48	28%	(22–34)	27%	(21–33)	22%	(16–27)	36%	(29–42)	28%	(22–33)
60	24%	(19–30)	24%	(19–30)	20%	(14–24)	30%	(24–37)	23%	(18–29)
Median	14 months	(11–17)	12 months	(10–16)	10 months	(8–12)	23 months	(18–33)	16 months	(12–19)
**Multifocal PSMs**										
12	37%	(23–50)	29%	(17–42)	23%	(12–36)	47%	(32–60)	38%	(24–50)
24	19%	(8–33)	35%	(19–51)	15%	(6–27)	31%	(17–46)	23%	(12–36)
36	15%	(5–29)	21%	(11–34)	7%	(2–20)	23%	(11–39)	17%	(10–33)
48	11%	(3–25)	6%	(1–17)	7%	(2–20)	12%	(8–35)	9%	(2–20)
60	7%	(1–20)	6%	(1–17)	3%	(0–15)	8%	(1–21)	6%	(1–16)
Median	8 months	(5–14)	7 months	(3–11)	5 months	(3–10)	9 months	(7–19)	8 months	(6–11)
**Soft Tissue PSMs**										
12	47%	(40–54)	43%	(37–50)	38%	(32–44)	58%	(51–64)	50%	(43–56)
24	32%	(25–38)	29%	(23–36)	25%	(19–31)	40%	(33–47)	33%	(26–39)
36	27%	(20–33)	23%	(16–30)	19%	(13–25)	35%	(27–42)	28%	(21–34)
48	23%	(17–30)	20%	(14–27)	18%	(12–24)	28%	(20–36)	22%	(16–28)
60	20%	(13–28)	19%	(13–26)	16%	(11–23)	25%	(18–33)	18%	(12–25)
Median	11 months	(9–14)	11 months	(8–12)	9 months	(6–11)	16 months	(13–21)	12 months	(10–16)
**Urethral PSMs**										
12	52%	(38–64)	54%	(40–66)	45%	(31–57)	68%	(53–79)	56%	(43–68)
24	42%	(29–56)	44%	(31–57)	38%	(25–51)	56%	(41–69)	43%	(30–56)
36	35%	(21–49)	37%	(24–50)	30%	(18–44)	48%	(33–62)	35%	(23–48)
48	29%	(16–43)	29%	(17–42)	24%	(13–38)	40%	(25–54)	29%	(17–41)
60	23%	(12–37)	26%	(14–40)	21%	(10–35)	30%	(17–45)	20%	(10–32)
Median	13 months	(7–30)	15 months	(5–37)	9 months	(5–27)	30 months	(14–53)	15 months	(6–30)
**Ureteric PSMs**										
12	66%	(48–73)	58%	(45–69)	51%	(38–63)	77%	(64–86)	55%	(49–60)
24	49%	(34–61)	52%	(38–64)	40%	(27–52)	67%	(53–78)	40%	(34–45)
36	37%	(23–51)	36%	(23–50)	27%	(16–40)	52%	(36–66)	33%	(27–38)
48	37%	(23–51)	34%	(21–47)	27%	(16–40)	46%	(30–61)	28%	(22–33)
60	34%	(20–48)	28%	(16–42)	22%	(11–35)	39%	(24–55)	23%	(18–29)
Median	23 months	(14–53)	30 months	(8–36)	14 months	(7–30)	39 months	(26-NR)	28 months	(18–51)

Abbreviations are as follows: RFS, relapse free-survival; MFS, metastasis free survival; CSS, cancer specific survival; OS, overall survival; RC, radical cystectomy; PSMs, positive surgical margins.

**Table 3:** Multivariable Cox regression analyses predicting oncological outcomes.

	Loco Regional RFS				MFS				RFS				OS			
	HR	95% Confidence Interval		*p*	HR	95% Confidence Interval		*p*	HR	95% Confidence Interval		*p*	HR	95% Confidence Interval		*p*
		Lower	Upper			Lower	Upper			Lower	Upper			Lower	Upper	
**Age at Diagnosis**	-	-	-	**-**	-	-	-	**-**	-	-	-	**-**	1.01	1.01	1.03	**0.015**
**Number of lymph nodes removed**	0.98	0.97	0.99	**0.022**	0.98	0.97	0.99	**0.007**	0.98	0.97	0.99	**0.007**	-	-	-	**-**
**Pathologic staging at RC**																
pT0-is-1–2	Ref				Ref				Ref				Ref			
pT3–4	3.32	1.84	5.99	**<0.0001**	2.80	1.66	4.70	**<0.0001**	2.63	1.56	4.46	**0.0003**	3.37	1.93	5.87	**<0.0001**
**Pathologic lymph node staging at RC**																
pN0	Ref				Ref				Ref				Ref			
pN1–2-3	2.14	1.49	3.08	**<0.0001**	2.60	1.86	3.63	**<0.0001**	2.43	1.72	3.44	**<0.0001**	1.83	1.32	2.53	**0.0003**
**Type of positive surgical margin**																
Soft tissue PSM	Ref				Ref				Ref				Ref			
Urethral PSM	1.03	0.62	1.71	0.909	0.90	0.53	1.55	0.72	0.92	0.55	1.52	0.75	1.08	0.67	1.72	0.74
Ureteral PSM	0.85	0.48	1.50	0.587	0.73	0.42	1.28	0.27	0.87	0.52	1.45	0.59	0.65	0.37	1.14	0.13
Multifocal PSMs	1.44	0.98	2.11	0.056	1.51	1.04	2.18	**0.02**	1.47	1.02	2.12	**0.03**	1.50	1.06	2.13	**0.02**
**Adjuvant chemotherapy**																
No	Ref								Ref				Ref			
Yes	0.58	0.40	0.84	**0.004**	0.65	0.45	0.93	**0.02**	0.59	0.41	0.83	**0.003**	0.58	0.40	0.83	**0.003**

Abbreviations are as follows: RFS, relapse free-survival; MFS, metastasis free survival; CSS, cancer specific survival; OS, overall survival; RC, radical cystectomy; PSMs, positive surgical margins.

## Data Availability

De-identified data from this study is stored by P.S.
